# Neural Membrane Signaling Platforms

**DOI:** 10.3390/ijms11062421

**Published:** 2010-06-10

**Authors:** Ron Wallace

**Affiliations:** Department of Anthropology, University of Central Florida, Box 25000, Orlando, FL, 32816, USA; E-Mail:rwallace@pegasus.cc.ucf.edu; Tel.: +1-407-823-2227; Fax: +1-407-823-3498

**Keywords:** microdomains, rafts, lipids, neurons, artificial intelligence

## Abstract

Throughout much of the history of biology, the cell membrane was functionally defined as a semi-permeable barrier separating aqueous compartments, and an anchoring site for proteins. Little attention was devoted to its possible regulatory role in intracellular molecular processes and neuron electrical signaling. This article reviews the history of membrane studies and the current state of the art. Emphasis is placed on natural and artificial membrane studies of electric field effects on molecular organization, especially as these may relate to impulse propagation in neurons. Implications of these studies for new designs in artificial intelligence are briefly examined.

## 1. Introduction

The plasma membrane of eukaryotic cells constantly mediates between internal and external environments. Consistent with its role as a regulatory boundary, the membrane is not a homogeneous liquid, as originally believed, but a richly compartmentalized dynamic structure with a wide range of functional properties. This review evaluates a rapidly growing body of evidence suggesting that microdomains or rafts may modulate membrane protein activity and thus function as signaling platforms. Microdomains are very small membrane regions (5–20 nm) with elevated concentrations of sphingolipids and cholesterol. (Caveolae, which are flask-shaped invaginations of the plasma membrane, are not discussed in this review. For a good recent overview of the topic see [1]). The smallest microdomains may contain as few as ∼30–40 lipids and ∼6–10 proteins. Their lifetimes tend to be short (1 millisecond or less), and their composition may vary due to molecules in the fluid environment diffusing in and out of the microdomains [2–4]. In the neuron, microdomain interaction with ion channels may play an important role in action potential (AP) propagation. The review begins with an historical overview of pioneering membrane studies, ranging from the 19^th^ century experiments of Henri Dutrochet which first suggested the membrane’s existence, to the 1972 Fluid Mosaic Model proposed by Singer and Nicolson. Subsequent studies of membrane order are then examined in some detail, methodological problems are discussed, and a revised understanding of neuron information-processing involving electrostatic interaction between ion channel charge groups and transient membrane dipoles is briefly presented. Finally, the possibility of membrane-based devices which simulate neuron microdomains is schematically evaluated. It is concluded that despite significant engineering obstacles, such devices would possibly have important implications for artificial intelligence.

## 2. Pioneering Studies: From Dutrochet to Singer and Nicolson

Membrane researchers in the early 19^th^ century energetically engaged the question of whether the membrane in fact existed [5,6]. The controversy was almost inevitable, given the instrumental limitations of the day including, in particular, the low-resolution microscopes. Thus when Henri Dutrochet in 1824 advanced the concept of a “globule” (cell) semi-permeable membrane, based on the uniform cell morphology he had observed in animal tissue, his view was strongly resisted by many of his contemporaries. Fallible instrumentation, however, was not the only issue. German cytologists, especially Max Schultze and Franz Unger, proponents of the “Membraneless State Model”, were impressed by phagocytotic movements observed in unicellular organisms. (Their opponents, the “Membran” school, argued for a rigid cell wall comparable to that of plant cells.) These dynamics seemed incompatible with the constraint of a membrane, and were attributed to “monads” characterized by “self-determination”. The latter view, clearly influenced by vitalistic philosophy, impeded membrane research until nearly the end of the century when the field was dramatically transformed by the work of Charles Ernest Overton.

As he addressed the October 31, 1898 gathering of the Science Foundation in Zurich, Charles Overton noted that for over nine years he had conducted some 10,000 experiments “on the general osmotic properties of plant and animal cells.” “I should have reached,” he said in deliberate understatement, “a satisfactory general view about them.” The experiments were models of scientific caution. Initially, he extended Hugo de Vries’ studies in osmosis by investigating cell permeability in solutions containing increasing quantities of a compound. Satisfied that the cell boundary was a semi-permeable barrier, he then turned to the problem of explaining the varied rates at which compounds entered the cell. He found that compounds soluble in fatty oils entered the cell very quickly while compounds soluble in water but “insoluble or very slightly soluble” in fatty oil would either penetrate the cell very slowly or would not penetrate it at all. He was ultimately led to the notion that the cell’s outer boundary—he was now using the phrase “precipitation membrane”—was “impregnated by a substance whose dissolving properties for various compounds may well match those of a fatty oil.” The concept of the lipid membrane as a selective semipermeable barrier was thus introduced to cell biology.

Overton’s inability to describe membrane structure in any but the most general terms reflected the persisting limits of instrumentation. Not until the development of electron microscopy in the 1930s and 1940s would it be possible to directly observe the lipid membrane *in situ*. Nonetheless, attempts to design a device for preparing thin films, or artificial membranes, were already underway eight years before the Zurich paper. Agnes Pockels, a pioneering amateur, had used “a rectangular tin trough filled with water to the brim” to study the surface tension of fluids. Her apparatus was modified in 1917 by Irving Langmuir and Katherine Blodgett and is now known, perhaps somewhat unfairly, as a Langmuir-Blodgett (LB) trough. Using the improved device, Langmuir and Blodgett demonstrated that lipids with varied hydrocarbon-chain lengths would always produce films with the same surface area. This result would only occur, they reasoned, if the lipid molecules were vertical. The most chemically plausible orientation would be for the polar head group to be in contact with the water, and the non-polar hydrocarbon chains pointing upward. Seeking to replicate this result, Edwin Gorter and F. Grendel in 1925 extracted lipids from erythrocytes of several different animal species and applied them to the surface of water in a modified LB trough. Their unexpected finding—only possible because their mathematical and procedural errors cancelled each other out—was that the area covered by the lipids was twice the total surface area of the erythrocytes. This result was only possible, they concluded, if the cell membrane were a bilayer: “One obtains a quantity of lipoids that is exactly sufficient to cover the total surface of the chromocytes [erythrocytes] in a layer that is two molecules thick.”

It is retrospectively somewhat surprising that these progressively accurate understandings of membrane structure were succeeded in the 1930s by a model both counterintuitive and historically retrograde. Hugh Davson and James F. Danielli proposed in 1935 that the membrane was a sandwich-like structure with protein surfaces and a lipid interior [7]. The Davson-Danielli (DD) model, reminiscent of the rigid cell wall (“Membran”) of 19^th^ century German cytology, was nearly as difficult to reconcile with membrane flexibility as its counterpart had been in the previous century. However, membrane studies conducted in the 1940s and 1950s using the newly-developed electron microscope initially appeared consistent with the model. The 1 nm resolution images revealed a trilaminar membrane structure of dark, light, and dark bands, often analogized to railroad tracks. The dark bands were believed to be the protein coats, while the light band was proposed to be the lipid interior. J.D. Robertson importantly modified the model in 1960 by demonstrating that the dark bands were headgroups and associated proteins of lipid monolayers, noting correctly that the lipid bilayer was a universal structural feature of animal cells [8]. With Robertson’s modifications, DD continued as the dominant model until a decisive experiment conducted in 1970 by L.D. Frye and Michael Edidin.

The central feature of DD, the protein “sandwich”, implied limited protein mobility in the plane of the bilayer. Frye and Edidin tested this feature by fusing mouse and human lymphocytes (heterokaryon assay) and then tracking the lateral mobility of major histocompatibility complex (MHC) proteins of the two species [9]. Within 40 min after fusion, they noted that “total mixing of both parental antigens occurred in over 90% of the heterokaryons.” Based on this observation, they proposed a major revision in the understanding of membrane structure: “It appears that the cell surface of heterokaryons is not a rigid structure, but is ‘fluid’ enough to allow free ‘diffusion’ of surface antigens resulting in their intermingling within minutes after the initiation of fusion.” Strongly influenced by this experiment (as well as by earlier studies conducted by Lenard and Singer indicating that labeled proteins form isolated spots in some membranes), Jonathan Singer and Garth Nicolson proposed the Fluid Mosaic model (FM) of membrane architecture in 1972, which has continued as the dominant model to the present day [10]. In FM, the membrane is construed as a 2D sea of lipids with a “mosaic” of integral proteins. Consistent with the fluid viewpoint, molecules comprising the membrane form random associations, resulting in overall lateral disorder. It is important to note, however, that Singer and Nicolson expressed reservations regarding the scale of disorder, noting that FM applied to “distances of the order of a few tenths of a micron and greater.” Their caveat left open the possibility of small-scale order, and ushered in an era of research on membrane lateral heterogeneities.

## 3. Membrane Small-scale Oder: Microdomains

**Figure 1.**
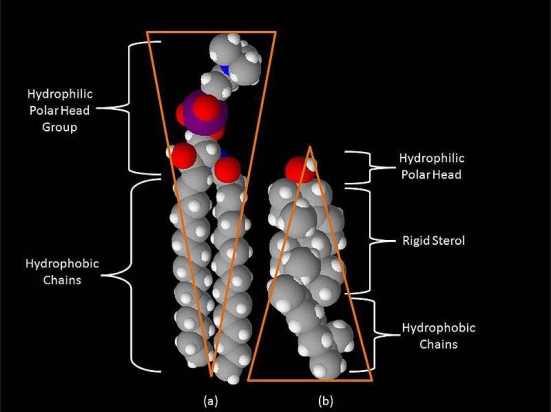
Space-filling models of sphingomyelin **(a)** and cholesterol **(b)**. Note the complementary cone-like shapes representing spaces occupied by hydrophobic and hydrophilic regions. Sphingolipids (such as sphingomyelin) and cholesterol form aggregates in the cell membrane known as microdomains or rafts. Ranging in size from 5-20 nanometers, with lifetimes of 1 millisecond or less, they play critical roles in cell signaling, including electrochemical signaling in the neuron.

It is testimony to the indirect pattern of scientific advance that evidence supporting small-scale membrane order actually preceded the concept of the bilayer as a 2D fluid. As early as 1965, a full seven years before FM, studies of the viscosities and heat capacities of organic liquids had revealed quasicrystalline molecular clusters in the liquid phase together with freely dispersed molecules. Motivated by these findings, A.G. Lee and his colleagues examined the effect of temperature change on lipid cluster formation in dioleoyllecithin bilayers [11] In keeping with earlier findings that the quasicrystalline clusters break up into monomeric, dispersed molecules, the investigators noted that clusters occurred in the membrane up to 30 °C. By 1978, following a series of similar experiments, it was widely recognized that membranes were typically comprised of “lipids in a more ordered state”. Shortly afterward (1982), Morris Karanovski’s group presented evidence of membrane heterogeneity using the probe 1,6-diphenyl-1,3,5-hexatriene (DPH), for which the lifetime decay is sensitive to the phase of the lipid environment [12]. Equal in importance to their demonstration of heterogeneity was a concluding series of questions which would echo throughout membrane research: 1) Are membrane proteins localized in lipid domains? 2) Do changes in the lipid environment modulate protein function? 3) Does cholesterol depletion make “gel-like domains” more fluid? And (conversely) does cholesterol replenishment restore the gel-like quality? A decade later, these queries took on fresh significance when Deborah Brown and J.K. Rose operationally defined the microdomain or “raft” (Figure 1). Earlier studies had shown that sphingolipids in the outer membrane leaflet constituted stable ensembles in which glycosylphosphatidylinositol (GPI)-anchored proteins were likely to be confined. Brown and Rose determined that the sphingolipids and GPI-anchored proteins were insoluble in cold detergent (Triton X-100), and floated to the top of a sucrose density gradient as a cholesterol-dependent fraction [13]. Their experiment (which was followed by many similar studies) became the basis for functionally defining a microdomain or “raft” as a detergent-resistant membrane (DRM) fraction.

Brown and Rose had introduced a major laboratory technique used to isolate microdomains. Yet as later studies refined the detergent-extraction method (e.g., using other detergents such as NP-40, octylglucoside, CHAPS, and lowered concentrations of Triton X-100) skepticism grew, and persists to the present time, regarding their relevance to living systems [14]. Although the various extraction methods yield microdomains with substantial overlap in protein and lipid content, there are significant differences as well, suggesting that microdomains may be the product of the method by which they are synthesized. Further complicating the issue, DRM fractions are low in anionic phospholipids and phosphatidylethanolamine, both of which are typically concentrated in the membrane inner leaflet. Does membrane detergent treatment selectively extract the exofacial leaflet and leave the inner leaflet behind? In an attempt to resolve these difficulties, there has been extensive use of an alternative biochemical technique—cholesterol depletion—to study microdomain properties. The rationale is straightforward: because microdomains, by definition, require cholesterol, its depletion should disrupt a wide variety of cellular functions believed to be microdomain-dependent. Depletion is generally accomplished through sequestration by cholesterol-binding compounds, removal from the membrane through methyl-beta-cyclodextrin treatment, or inhibition of cholesterol biosynthesis. But controversy persists. The method significantly alters cell morphology, exocytosis, and intracellular signaling; moreover it disrupts the actin cytoskeleton, a perturbation which could, in turn, produce an array of pleiotropic effects. In response to these critiques, proponents argue that carefully controlled partial cholesterol depletion (∼50%) from the outer membrane leaflet can produce acute effects within approximately ten minutes without significantly affecting other cellular processes. Perhaps all that one may safely say of a rapidly intensifying debate in which, all too frequently, methodological claims are based on a limited number of studies, is that all the biochemical techniques perturb the membrane to some extent, and the strongest claim to be made (so far) is that the damage is localized. As a consequence, biophysical methods which are sensitive to membrane dynamics while protecting the integrity of the bilayer are increasingly being utilized.

The biophysical techniques can reveal microdomain properties at previously inaccessible levels of temporal and spatial resolution [15,16]. One of the most widely used techniques is fluorescence (or Förster) resonance energy transfer (FRET), in which proteins of interest are labeled with donor and acceptor fluorophores. When the labeled molecules are within ∼100 Å of one another, energy transfer occurs. Transfer is normally detected and quantified by monitoring changes in the fluorescence of the donor in the presence or absence of the receptor. In a variation of the technique known as homoFRET, changes in the polarization of fluorescence are monitored when the same type of fluorphore serves as donor and acceptor. The application of FRET has generated high-resolution images of GPI-anchored protein clusters of 4–5 nm radius. More generally, these “snapshots” depict pronounced spatial heterogeneity of inner and outer bilayer leaflets, consistent with current thinking regarding the liquid-ordered state of the membrane. Also in keeping with this viewpoint is the hypothesis that membrane protein diffusion should be slowed within the plane of the bilayer due to either transient or permanent microdomain association. This possibility has been examined through fluorescence recovery after photobleaching (FRAP). In this technique, a population of molecules within a membrane region is uniformly labeled with fluorescent tags. The region is then photobleached with a high-intensity light pulse, causing the fluorescence lifetime of the target molecules to quickly elapse. The resulting optical effect is a uniformly fluorescent field containing a noticeable dark spot. As the region is monitored, the still-fluorescing probes will diffuse throughout the sample, replacing the non-fluorescing probes in the bleached area. The speed of fluorescence recovery is thus a measure of the fraction of membrane molecules in the region that are capable of diffusing freely (the mobile fraction). FRAP studies have indicated that, under steady-state conditions, microdomain-associated proteins will display relatively rapid lateral diffusion through the membrane, suggesting they are dynamic structures. However, because the approach is a population-based measurement, it cannot provide detailed information regarding molecular mechanisms underlying the diffusion process. Thus for a more complete picture of microdomain dynamics, FRAP observations are perhaps best interpreted against the backdrop of single-particle tracking (SPT) studies, which follow the behavior of individual protein and lipid molecules.

The SPT method, which is used with living cells, involves the labeling of selected individual protein or lipid molecules with fluorophores (e.g., green fluorescent protein, cyanine dye) or with probes that are visible with transmitted light (gold or latex beads). Movements of the labeled molecules are then monitored on nanometer spatial and sub-millisecond temporal scales. SPT has been extensively used to study the diffusion of a variety of membrane proteins, including Lck (an SRC family protein tyrosine kinase), synaptic receptors for glutamate and glycine, and the cystic fibrosis transmembrane conductance regulator. Through improvements in the technique, it has been possible to address unresolved difficulties in the FM model, recently resulting in a novel viewpoint which may arguably be designated a “paradigm shift”. Akihiro Kusumi’s Membrane Organizer Project has challenged FM through high-speed tracking of an unsaturated phospholipid *L*-α-dioleoylphosphatidylethanolamine (DOPE) labeled with a colloidal gold particle [17]. In FM, as their study notes, there had never been an adequate explanation of why the diffusion coefficients for both proteins and lipids in the plasma membrane were smaller (by factors of 5 to 50) than in artificially reconstituted membranes. In principle, if both types of membrane were comprised of a “sea of lipids”, the diffusion coefficients should not have differed significantly. Moreover, there was the problem of molecular complexes, or oligomers: When these units (which typically included receptors and other signaling molecules) were monitored in plasma membranes and artificial membranes, the reduction of the diffusion coefficient in the former was similarly dramatic. Indeed, in some instances, there was temporary immobilization. (The latter finding also violated the formal prediction of an equation developed in 1975 by P.G Saffman and M. Delbrück that the formation of molecular complexes in the lipid bilayer should not significantly reduce the diffusion rate [18]. The contradiction is unsurprising, however, when one reflects that their equation was formulated on the basis of the Singer and Nicolson model.) Importantly, these questions now appear to have been resolved by the Kusumi studies. The investigators found that the plasma membrane is partitioned into submicron compartments (“corrals”) comprised of actin cytoskeleton “fences” anchored by transmembrane protein “pickets”. The compartments or corrals, which vary in size from 30 nm to 230 nm, are possibly the result of ∼3 billion years of cellular evolution in which there would have been strong natural selection pressure for a physical mechanism to regulate the long-range diffusion of membrane molecules. In the new viewpoint, membrane monomers hop across the “picket fence” (hop diffusion) “with relative ease”, while molecular complexes are more likely to be tethered to the membrane skeleton (oligomerization-induced trapping). In a variation of this model, originally proposed by A. Tsuji and S. Ohnishi, monomers move through cytoskeleton “gates” created by the dissociation of the spectrin tetramer into two dimers (open state) and subsequent re-association (closed state) [19,20]. Monte Carlo simulations based on the latter interpretation suggest that different gate-closing rates may carefully regulate inter-compartmental diffusion and (in the case of the faster closing rates) confine monomers within a corral [21–23]. In addition to possibly explaining the difficulties with FM, the Kusumi model (and its variant) may have important implications for cellular signaling. Oligomerization-induced trapping in a cytoskeleton corral could in principle stabilize a set of signal-protein co-factors localized in a microdomain. This feature, which is probably important in all types of cell membranes, may be of particular significance in neuron signaling systems.

## 4. Neural Membrane Microdomains as Signaling Platforms

From the standpoint of information theory, any signal is a physical structure for which temporal or spatial changes of state encode information, or recognized contrast with the environment. In the neuron, signals include ligand- and voltage-gated ion channels, G-protein-coupled receptors, receptor tyrosine kinases, and a variety of other molecular factors. The simplest function of a microdomain is to provide a scaffold or platform to co-localize the molecular signals, thus facilitating their interaction [24–26]. For example, a neuron receptor could be localized only to those microdomains which contain a specific set of signal components, thereby preventing or significantly limiting non-specific signaling. A more complex possibility exists: In a microdomain-based switching sytem, the components necessary to activate or modulate a signaling pathway could initially be segregated into separate microdomains. Subsequent microdomain fusion, induced by an external input, could activate or refine the signal. The separation of the microdomains, following the termination of the input, would then re-set the system. It is important to note that in both types of processes, microdomains are essentially passive, providing platforms for proteins. Is it possible that microdomains may also play a more active role, modulating by means of their intrinsic chemical structure intracellular traffic and electrical signaling in the neuron? In this section, we review recent studies of neuron microdomains, and evaluate this possibility.

### 4.1. Neural Microdomains, Development, and Plasticity

Viewed from an adaptive standpoint, the highly dynamic environments of the more behaviorally complex species (especially social carnivores, nonhuman primates, and humans) require constant structural changes in their information-processing machinery. As a result, many—if not all—of the intracellular molecular systems which regulate changes in neural structure and network connectivity from embryo to maturity continue to operate throughout adult life, thereby blurring the conventional distinction between development and plasticity. Moreover, because some of these systems mediate between electrical or molecular inputs and the information encoded in genes, they are of considerable theoretical importance in the biology of human behavior [27]. Increasing evidence suggests that microdomains may play a major role in activating or modulating these pathways. One striking example is neurotrophic-factor, or neurotrophin, signaling.

**Figure 2.**
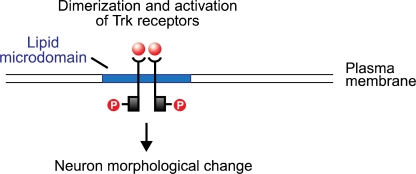
Microdomain (raft) scaffolding permits stable co-localization of molecular signals, thus facilitating their interaction. Neurotrophin binds as a dimer to the microdomain-stabilized Trk receptor protein. The Trk receptor then dimerizes, which initiates a complex chain of phosphorylation events resulting in neuron morphological change. Illustration adapted from [31].

Neurotrophins are polypeptides critical to the growth, differentiation and survival of immature neurons, as well as to the maintenance of neurons in the adult nervous system. One of the best-understood neurotrophins in neuron growth factor (NGF), extensively studied for sixty years. Historically, NGF played a significant role in Victor Hamburger and Rita Levi-Montalcini’s formulation of the Neurtrophic Factor Hypothesis (NFH) [28,29]. The investigators demonstrated that the size of a developing neuron could be modified by changing the size of its innervation target. They explained this result by proposing a “metabolic exchange between the neurite and the substrate to which it grows”. Implicit in NFH was the concept that neurotrophins, synthesized and secreted by the target neuron, bind with receptors on the innervating neuron in a form of retrograde signaling. Subsequent studies of NGF and the other neurotrophins, Brain-Derived Neurotrophic Factor (BDNF), NT-3, NT-4/5, and NT-6, have strongly supported NFH [30]. The neurotrophins bind to the tyrosine kinase (Trk) receptors TrkA, TrkB, and TrkC, and to the low-affinity glycoprotein receptor p75. (The last, a tumor necrosis factor, is structurally unrelated to the Trk proteins, and appears to potentiate the pathway activated by NGF binding with TrkA.) The neurotrophin molecule binds as a dimer, and in turn causes the dimerization of the Trk receptor (Figure 2). The cytoplasmic tyrosine kinase domains on the polypeptide chains of the Trk dimer then cross-phosphorylate, creating docking sites for the src homology domain 2 (SH-2), present on several cellular proteins. One of these is Shc which, following binding with the phosphotyrosine region of the Trk receptor, is itself phosphorylated by Trk. Shc binding and activation initiates a cascade of phosphorylation events, the first stage of which terminates with the exchange of GDP for GTP on the ras protein. Ras, which is active in its GTP-bound form, initiates a second phosphorylation sequence terminating in the activation of the Extracellular Response Kinase (ERK) protein. ERK initiates two pathways, both of which are significant to neuron modification: one is a phosphorylation sequence involving numerous cellular proteins including tyrosine hydroxylase and microtubule proteins; the other is the entry of activated ERK into the nucleus where it interacts with transcription factors that regulate the rate of mRNA synthesis.

Although much is still unknown regarding the final stages of neurotrophin-induced signaling—e.g., the precise sequences that link ERK activation with neuron morphological change—two conclusions may be reached regarding the neurotrophin-Ras-ERK pathway. One is that microdomains are clearly crucial for effective neurotrophin signaling [31–33]. As noted above, a fundamental function of the microdomain is a scaffold or platform which co-localizes molecular signals, thereby facilitating their interaction. TrkA and B are enriched in microdomains, which enhances autophosphorylation following neurotrophin binding. Following cholesterol depletion with cyclodextrin, Trk autophosphorylation is disrupted by >50% (but see the cautionary discussion above). Also, many intermediates in the Trk cascade are microdomain-associated, including Shc and ras. Perhaps the latter finding exemplifies the separation of signal components into separate microdomains when the initial signaling molecule (Trk) is not activated, followed by microdomain fusion after neurotrophin binding. This, and other features of microdomain-modulated intracellular signaling, requires further investigation. The second conclusion that may be drawn is that neurotrophin signaling occurs in the adult brain, thus illustrating the fading distinction between development and plasticity. James Conner’s team recently demonstrated that NGF infusions into the adult rat hippocampus enhanced long-term potentiation (LTP), an electrophysiological correlate of neural plasticity [34]. Conversely, NGF blockade through infusion of an NGF antibody, significantly reduced LTP and impaired spatial learning. In view of separate evidence that other cognitive functions, including novelty detection, are localized in the hippocampus, it would be of interest to determine if these were modifiable through NGF augmentation and blockade.

In contrast to neurotrophin signaling, the molecular sequences involved in axon elongation and targeting are less well understood. There is as yet only limited evidence suggesting that the response of the neural growth cone, the region at the leading tip of the neurite, to an extracellular diffusion gradient of molecules produced and released by a target neuron may be microdomain-mediated. Growth-cone navigation is initiated by the binding of the extracellular cues (comprising the diffusion gradient) to plasma membrane receptors, followed by receptor oligomerization and complex formation with co-receptors or other signal proteins [35–37] Plasma membrane complex formation in turn stimulates an intracellular cascade resulting in cytoskeleton re-organization which transforms neurite morphology. The precise targeting which is essential for constructing a neural network is achieved by spatial asymmetry of the plasma membrane signaling complexes. Anterograde migration is generated by a shift in receptor activation toward the front of the growing cell, while neurite turning results from shifts in activation to the left or right. These changes are in turn converted into the activation and inhibition of intracellular signaling pathways which ultimately regulate navigation. A few key studies suggest that microdomains may be essential scaffolds for concentrating the signaling molecules. In a recent set of experiments conducted by Carmine Guirland’s team, microdomains in developing neurons were selectively disrupted by the application of methyl-β-cyclodextrin (MCD) which depletes membrane cholesterol (see earlier methodological discussion), and the effect on navigation was monitored by light microscopy [38]. As a precaution against changes in neurite mobility being an artifact of chlolesterol depletion, microdomains were also disrupted using filipin, which disrupts microdomains without extracting cholesterol. The results strongly suggested an important role for microdomains in neuron development. Neuron responses to diffusion gradients of several extracellular cues, including BDNF, neutrin-1, and Semaphorin 3A, were blocked by disrupting microdomain integrity. In a similar vein, Hiroyuki Kamiguchi’s group inactivated microdomains by fluorescein-labeling a GMI ganglioside (contained in the microdomain outer leaflet) which in turn releases singlet oxygen upon laser irradiation [39]. The disruptive effect of the singlet oxygen is highly focal, extending for only 5 nm. Consistent with Guirland’s results, the response of the growth cone tip to N-cadherin and L1, two cell adhesion molecules (CAMs) was blocked by microdomain disruption. Together these data suggest that, while many features of the process are still imperfectly understood, it is becoming evident that microdomains are essential to axon targeting during brain development. Important questions for future research include the identification of the specific intracellular pathways involved, the mechanics of pathway cross-talk, and the extent to which these cascades play a role in adult plasticity.

In pursuing the latter questions, the relatively well-defined interactions involved in activity-dependent signaling to the nucleus via cyclic AMP response element binding protein (CREB) may possibly serve as a prototype. Current models of the CREB pathway specify, to a relatively complete extent, a regulatory system that begins with cystolic Ca^2+^ elevation, extends to CREB phosphorylation, and terminates with evolutionarily-related behaviors such as spatial mapping, fear conditioning, and circadian activity [40–42]. The system is therefore important as a paradigmatic example of gene-brain reciprocal signaling in the biology of human behavior [43. 44]. Cholesterol-depletion experiments which disrupt downstream pathways (but see earlier cautionary remarks regarding this method) suggest that Ca^2+^ entry into the cytosol, which initiates the CREB cascades may be stabilized by microdomain association and clustering of signal components. L-type voltage-sensitive Ca^2+^ channels (L-VSCCs), the ligand-gated Ca^2+^-conducting N-methyl-D-aspartate (NMDA) glutamate receptor channel (co-localized wth the Ca^2+^-conducting α-amino-3-hydroxy-5-methyl-4- isoxazolepropionic (AMPA) channel), and the epidermal growth factor (EGF) tyrosine kinase receptor involved in Ca^2+^ release, are all microdomain-localized. Biswaranjan Pani and Birj Singh, in a recent review [45], propose that microdomains stabilize these signal components, a view consistent with Kusumi’s oligomerization-induced trapping model discussed above. This view appears particularly plausible in the relation to the NMDA and AMPA receptor channels, in that intensive glutamate activation of AMPA must coincide with glutamate binding with NMDA to remove NMDA channel blockade by extracellular magnesium, permitting Ca^2+^ influx. This “coincidence detector” function would likely not be possible if the receptors were not co-localized by means of microdomain stabilization. An alternative modality is Ca^2+^ release from endoplasmic reticulum (ER) stores. Hormone or growth-factor binding with microdomain-associated tyrosine kinase receptors initiates a signal cascade which generates inositol 1,4,5 triphosphate (IP_3_). IP_3_ in turn binds with ER-localized IP_3_ receptors, accomplishing Ca^2+^ release from the intracellular stores. In an intriguing—and pioneering—discussion, Pani and Singh propose that co-localization of the Ca^2+^-conducting ER TRPC channels and the ER STIM1 proteins which regulate them, is facilitated by ER microdomain scaffolding. If their model turns out to be correct, it would suggest that microdomain localization is essential for spatio-temporal coordination of protein dynamics on both the organelle and plasma-membrane levels.

The series of events following Ca^2+^ elevation includes several mechanisms for Ca^2+^-CREB-gene signal amplification and damping. (An important, if daunting, question for future research is the relative strength of each mechanism’s contribution to the final behavioral phenotype.) A critical early event is Ca^2+^ binding with the calmodulin protein (CaM). The latter may be regarded as a node in a molecular network which, given its multiple targets, could have an amplifying effect. CaM targets the protein kinases CaMKI, CaMKII, and especially CaMKIV, each of which phosphorylates CREB at a serine site (Ser-133), an event required for CREB function. The CREB protein, which is pre-bound to the CREB response element (CRE) promoter, is then enabled to recruit the CREB-binding protein (CBP) co-activator, which generates transcription activity. In addition to the Ca^2+^ cascade, CREB is activated through other important pathways including those initiated by neurotrophin binding with Trk receptors (see earlier discussion). Cross-talk between these pathways is another probable means by which input to CREB, and consequent up- or down-regulation of gene transcription, may be rapidly effected. A final modulatory mechanism which has only recently come to light involves modification of both CREB and CBP following Ser-133 phosphorylation and CBP recruitment. At this stage of the cascade, CREB may be additionally phosphorylated at Ser-142 and Ser-143 sites while CBP may be phosphorylated at Ser-301, and methylated at an arginine residue. As Bonnie Lonze and David Ginty have suggested, it is plausible that maximal phosphorylation may be associated with maximal gene expression [46]. Building upon that premise, the various modulatory mechanisms appear consistent with the concept of gene-brain cross-talk facilitating adaptation in complex, changing environments. Perhaps not surprisingly, brain systems currently identified with Ca^2+^-CREB-gene signaling are neural substrates for ancient behaviors—circadian activity, spatial mapping, fear conditioning, memory of defeat in aggressive encounters—for which environmentally-sensitive plasticity coupled with genome stabilization would have been essential for species survival. A full discussion of these systems is beyond the scope of this review. However, we may note that the final impression one takes away from these intricate, linked, and graded intracellular pathways is that of a hierarchy of evolutionarily optimized molecular regulators, at the apex of which is the neural membrane microdomain.

### 4.2. Microdomains, Action Potential Propagation, and Synaptic Transmission

In contrast to the intensive research on microdomain involvement in intracellular signaling, there has been relatively less emphasis on the possible role of these structures in impulse propagation. The disparity is somewhat surprising, given the strong historical priority of research on the latter topic. According to the conventional model formally described in 1952 by Alan Lloyd Hodgkin and Andrew Huxley, and based on their experiments with the squid giant axon, the neural impulse or action potential (AP) is initiated and propagated by means of trans-membrane ion flux through protein channels which successively generates, along the axon, a reversal of membrane potential at each channel locus above a critical threshold, thus relaying the impulse [47]. Nearly six decades later, the Hodgkin-Huxley (HH) model, although subjected to occasional critiques, remains the widely accepted explanation for electrical signaling in the neuron. Moreover, it has strongly influenced formal mathematical modeling of the artificial neuron, to be discussed briefly in the following section. Although microdomain stabilization of ion channels and channel co-factors is clearly relevant to HH, there is at the time of this writing only one major study of microdomain modulation of ion-channel properties. Jeffrey Martens’ team investigated the effect of cholesterol depletion on the electrical properties of the microdomain-associated potassium channel Kv2.1 [48,49]. The study determined that cholesterol depletion by cyclodextrin caused a ∼36 mV hyperpolarizing shift (from 15.7 [control] to −51.6 [cyclodextrin-treated]) in the midpoint of the Kv2.1 inactivation curve. Importantly, cyclodextrin treatment of the non-microdomain-associated channel Kv4.2 showed no observable effect on channel properties. The latter finding suggests that the modulation of Kv2.1 inactivation was due to the effect of cyclodextrin on the channel’s microdomain environment (including microdomain-associated signal proteins such as Trk), and not to damage to other cellular structures (e.g., the cytoskeleton). The study notes that Kv channels often contain phosphorylation sites, and that Kv2.1 in particular is tyrosine-phosphorylated. The observed inactivation effect may thus have resulted from cyclodextrin-induced separation of scaffolded signal components rather than from disruption of direct-microdomain-lipid interaction with the ion-channel protein.

The Martens experiment, like most of the studies in this review, emphasizes the scaffolding function of microdomains. Are there also intrinsic microdomain physical and chemical properties that modulate the activity of neural-membrane integral proteins, including in particular ion channels? To date, the answer is largely conjectural, suggesting a need for further research. Computer-simulation studies conducted by Harry Price and the author suggest that electric-field effects generated in the plane of the membrane during AP propagation may transiently alter microdomain physical properties in a manner that could modulate ion-channel activity, thereby altering neuron signaling [50,51]. Studies conducted with both natural and artificial membranes indicate that an applied electric field can produce electrophoretic movement of membrane proteins, and re-organize the bilayer [52–54] Concanavalin A (conA) protein receptors in living cells were redistributed in response to a 4 V/cm electric field, and phase separation of a two-component supported membrane was observed following the application of a 10–30 V/cm field. These data, and the results of similar experiments, are to be compared with the field strength of 100 kV/cm generated in the membrane at the peak of the AP.

On the basis of these and similar studies, we simulated the possible mechanical and electrostatic effects of an applied field on different membrane-lipid species. In our initial study, we investigated how the alignment of unsaturated bonds of model compounds would affect field-responsive properties (dipole and quadrupole moments, and polarizability). The results of our first model indicated a strongly increased sensitivity to field gradients as the number of unsaturated bonds is increased. In relation to this finding, a second, more complex, study investigated energetically-favorable clustering, and polarizability values, of model lipids (including, importantly, sphingolipids). It was found that sphingolipids display an energetically-favorable alignment of unsaturated bonds. (In an actual neuron, the alignment would be in the plane of the bilayer). In addition, the aligned bonds of the sphingolipids displayed the highest polarizability of all model lipid clusters. We then intuitively proposed that, in a neuron, transient dipoles would be generated in the clustered sphingolipids of an ion-channel associated microdomain as a field effect of channel gating and the trans-membrane current. We further suggested that the lipid dipoles would interact electrostatically with charge residues known to be present in an unfolded (random coil) ion-channel protein. Ion-channel closing (*i.e.*, a return to the α-helix conformation) could therefore be regarded as many-particle system seeking a minimum local potential energy. Put differently, the duration of the ion-channel “open” state, and hence the membrane potential, would be a function of the duration of lipid-protein electrostatic interactions in an ion-channel-associated microdomain. It would be, of course, incautious to infer too much from a limited number of studies. But the data appear to suggest that transient electrostatic interactions between a microdomain’s sphingolipids and an associated, voltage-gated, ion-channel protein may modulate AP propagation. Recalling Martens’ data, one might envision a variation of Stephen Waxman’s “multiplex” neuron in which an array of communicating modules with distinctive physical properties (electrostatics, phosphorylation) play specialized informational roles [55]. This possibility will be examined in more detail in the discussion of artificial intelligence.

The final stage of AP propagation is the relay of impulses into the neuron terminal, an event which generates intricate but temporally- and spatially-coordinated synaptic-transmission processes that are the basis of inter-neural signaling. In outline, synaptic transmission consists of the exocytosis of transmitter substance from lipid vesicles transiently fused with the pre-synaptic-terminal plasma membrane; the exocytosed neurotransmitter binds with post-synaptic receptors, resulting in target membrane depolarization, and continued AP propagation. Increasing evidence suggests that certain features of exocytosis may be microdomain-regulated [56–58]. It is important to note, however, that several aspects of the process are not well understood, and the richness of scenarios has occasionally surpassed that of the data. Given this caveat, it is clear that transmitter release is initiated by the influx of Ca^2+^ ions through microdomain-associated, voltage-gated channels in the presynaptic terminal plasma membrane [59]. Calcium ion elevation accomplishes the translocation of vesicles from an interior reserve pool to a “readily-releasable pool” (RRP) in the active zone of the pre-synaptic terminal, where they are docked and primed before fusing with the plasma membrane and releasing their transmitter cargo. Vesicle movement from the interior pool to the RRP is mediated by synapsin. This phosphoprotein is vesicle-associated and bound to actin; the latter may provide a scaffold for interior-reserve vesicles. Synapsin is phosphorylated by several different kinases (including CaMKII and protein kinases A and C), which causes it to dissociate from the interior reserve pool and translocate to the active zone of the presynaptic terminal plasma membrane (docking stage). It is estimated that ∼10–30 vesicles are docked at any one time, and that the majority are incapable of transmitter release; *i.e.*, the docked vesicles require maturation, or priming. The steps involved in the latter process remain somewhat undefined, but it is possible that the vesicles may partially fuse with the plasma membrane, and that the energy derived from ATP hydrolysis may alter the conformation of proteins comprising the exocytic machinery. Fusion, the following stage, is triggered by Ca^2+^ ions binding with negatively-charged aspartate residues (C2A and C2B) in a vesicle-associated protein sensor, synaptotagmin. Synaptotagmin may undergo an electrostatic rather than conformational change, causing it to interact with an elaborate vesicle-and plasma-membrane-associated protein machinery—the much-analyzed SNARE complex—that directly induces membrane fusion and transmitter exocytosis.

On purely intuitive grounds, it would seem that SNARE complex function would require microdomain localization of its protein components for rapid assembly, coordinated function, and disassembly. But artificial-membrane and *in vivo* membrane data are highly inconsistent, contrasting noticeably with the greater convergence in the SNARE-protein imaging studies [60–62]. SNARE (soluble N-ethylmaleimide-sensitive fusion protein (NSF) attachment protein (SNAP) receptor) association with microdomains has been documented for Madin-Darby canine kidney cells, 323-L1 adipocytes, HeLa cells, and rat brain somatosomes. However, artificial-membrane studies of the SNARE components syntaxin 1 (plasma membrane-associated SNARE) and synaptobrevin 2/VAMP2 (vesicle-associated SNARE) indicated a preference for the liquid-disordered phase. Interpretations of microdomain modulation of exocytosis are thus an uneasy blend of consensus and speculation. Based on X-ray crystallographic, FRET, and atomic force microscopy (AFM) studies, it is clear that the SNARE complex is formed by a zippering process, proceeding from N- to C-termini of the SNARE domains, in which the SNARE vesicle component synaptobrevin 2/VAMP2 is progressively tethered to the plasma-membrane SNARE components syntaxin and SNAP-25. Importantly, zippering/tethering is only possible when the Munc-18 protein separates from syntaxin, indicating a switching function. The role of microdomains in subsequent vesicle and plasma membrane fusion has been the subject of much speculation. A provocative set of models proposed by Christine Salaün’s team attempts to reconcile some of the conflicting data by proposing various ways in which microdomains *and* liquid-disordered domains would segregate different types of exocytosis [63]. For example, liquid-disordered domains would be the site of “kiss-and-run” exocytosis, in which a partial fusion of vesicle and plasma membranes creates a transient aqueous pore through which transmitter is released; alternatively, microdomains would be the site for full fusion of the two membranes. In what is clearly a programmatic article, the exact opposite scenario is also proposed, and other models are suggested as well. When exocytosis is complete—by means of one or several of these mechanisms—the NSF protein binds with SNAP-25 and, via energy derived from ATP hydrolysis, dissociates the SNARE complex for another exocytic cycle.

Exocytosed transmitter substances bind with a wide variety of post-synaptic receptors. It is becoming increasingly clear that microdomain and liquid-disordered membrane regions help regulate receptor function by alternately segregating and co-localizing receptor signal components; moreover, microdomains may also play an active role in receptor conformational changes essential for transmitter binding [64–67]. These functions have been investigated in a number of prototype systems, especially AMPA, acetylcholine, and serotonin. The AMPA receptor, as noted briefly above, functions together with the NMDA receptor in a “coincidence detector” mechanism; their interaction is a molecular substrate for long-term-potentiation (LTP), the neurophysiological basis for learning. A full discussion of LTP’s molecular pathways is beyond the scope of this review. Essentially, the AMPA and NMDA receptors are activated by glutamate, the most abundant neurotransmitter in the central nervous system. Glutamate binds with both receptor types, but opens only the AMPA receptor (causing Na^+^ influx) due to Mg^2+^ occlusion of the NMDA receptor channel pore. Subsequent depolarization of the AMPA receptor causes the repulsion of Mg^2+^, allowing Ca^2+^ influx. Calcium entry in turn generates a cascade of LTP-associated events. These include the upregulation of AMPA receptors to the membrane, and AMPA receptor phosphorylation by CaMKII, which increases its channel conductance. Recently, Qingming Hou’s team demonstrated microdomain localization of AMPA receptors by fluorescence imaging of double-labeled AMPA receptors and microdomains in cultured hippocampal neurons [68]. Additionally, their study determined that microdomain localization of AMPA receptors in cultured cortical neurons was increased by NMDA receptor activity, an effect which was abolished by an NMDA antagonist. The authors of the study propose that NMDA receptor activity may recruit AMPA receptors to microdomains, although the mechanisms remain uncertain. Once recruited, AMPA receptors may be phosphorylated by microdomain-associated Trk, possibly altering AMPA receptor activity levels and thereby modulating synaptic plasticity.

Hou’s research strongly suggests a microdomain scaffolding function which accomplishes the co-localization of AMPA and NMDA receptor signaling components. But is it possible that microdomains affect receptor dynamics in a more direct way? Jacques Fantini and Francisco Barrantes, in a recent review of receptor-microdomain interactions [69], propose that cholesterol and sphingolipids “are active partners of synaptic transmission.” Their model bridges several orders of magnitude, linking the physical chemistry of the acetylcholine and serotonin receptors with the regulation of transmitter binding and signal transduction. In the case of the acetylcholine receptor, a wide variety of imaging studies indicate that its microdomain environment is subdivided into bulk and shell lipids, both in a liquid-ordered phase. The shell component immediately surrounds the receptor, restricting its mobility for possible protein-cholesterol interaction. Although a cholesterol-binding motif has not yet been clearly identified for the acetylcholine receptor, several studies have shown that the receptor’s ordered α-helical structure and, importantly, ligand-binding stability, are both cholesterol-dependent. Sphingolipids may also be essential to effective acetylcholine-receptor function. Structural studies of a wide range of proteins indicate a sphingolipid binding domain (SBD), comprised of amino acid residues which bind to the polar headgroup of the sphingolipid. Based on these data, the investigators propose a ternary complex in which “cholesterol interacts simultaneously with one of the transmembrane domains of the receptor and with a glycosphingolipid.” The conformation of each of the three would be influenced by their cross-talk, a dynamic which in turn may regulate receptor sensitivity to neurotransmitter binding. Similarly, ligand binding and intracellular signaling of the G protein-coupled serotonin receptor depends upon microdomain integrity; receptor functions are disrupted by sphingolipid and/or cholesterol depletion, and recovered by its restoration. Based upon these data, and recent infrared and ultraviolet spectroscopy evidence for eight low-energy conformational isomers of serotonin [70], the investigators propose that interaction between cholesterol, sphingolipids, and the G protein-coupled serotonin receptor may regulate ligand binding and receptor-mediated signaling. In effect, the interactions within the ternary complex would “mold” the highly flexible serotonin molecule into an isomer specific for a serotonin-receptor subset. Microdomain sphingolipids, which display remarkable conformational variety (numbering in the hundreds of shapes) due to chain length, degree of saturation, and head-group structure, would play a determinative role in fashioning a distinctive shape for the serotonin molecule.

The computational implications of the Fantini-Barrantes model are worth considering. In essence, their viewpoint appears consistent with a concept of sub-neural information-processing modules in which the tokens are a set of interacting molecules seeking a minimum local potential energy. Receptor-channel gating and subsequent field-induced lateral movement of sphingolipids between post-synaptic microdomains, possibly by hop diffusion over cytoskeleton barriers, would modulate the computation, thus amounting to a form of learning. Possibilities such as these reflect the growing conceptual richness of microdomain theory, now entering its third decade. They also suggest a role for molecular-machine mimics in extending current models of artificial intelligence. This strategy will be discussed briefly in the following section.

## 5. Microdomain-based Molecular Machines and Artificial Intelligence

This review has evaluated a rapidly growing body of evidence suggesting that neural-membrane microdomains may function as dynamic scaffolds which co-localize signal units, thereby modulating neuron electrochemical information. These data, if reinforced by future investigations, may have important implications for the understanding of how the neuron works. In an admittedly speculative vein, but consistent with available data, it is here proposed that the neuron is not the fundamental unit of brain information-processing; rather, the neuron is more accurately regarded as a linear array of microdomain computational modules which can propagate, amplify, and indeed extinguish an AP, based on moment-to-moment changes in lipid composition and lipid-protein interactions [71]. As well, microdomains may up- or down-regulate intracellular molecular signals including, importantly, signaling to the nucleus. This approach is clearly at variance with the foundations of artificial intelligence. Over 60 years ago, Warren McCulloch and Walter Pitts, inspired by the symbolic logic of philosophers Alfred North Whitehead and Bertrand Russell, proposed an idealized neuron [72]. In their mathematical model, the neuron computed a weighted sum of inhibitory and excitatory inputs with regard to a threshold value, fired (if inputs were at or above threshold) in an all-or-none fashion, and propagated an invariant signal to another target unit. This view, which has given rise to many intellectual descendants—including the units in neural-network, perceptron, and connectionist theory—remains the key mathematical concept underlying formal modeling and physical implementation of neuropsychological processes. Yet, as several researchers have noted (based largely on extensive evidence for signal modulation in dendrites, as well as groups of synapses that depress nearest-neighbor activity) the neuron may not be analogous to a gate composed of a single transistor [73–75]. Instead, consistent with the viewpoint presented in this article, the neuron is more properly analogized to a chip or integrated circuit comprised of interconnected transistors.

**Figure 3.**
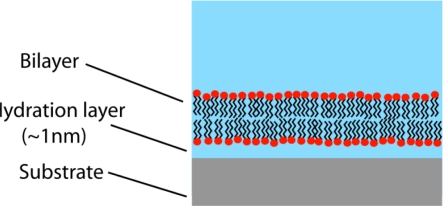
Supported lipid bilayer (highly schematic). Supports are typically gold or silicon. The membrane may be decoupled from the support by a water layer (shown) or a hydrated polymer. For stabilization, the latter structure is typically attached to the membrane by anchor lipids, and covalently tethered to the substrate. Functioning ion-channels can be inserted into the membrane. An important research goal is the synthesis of increasingly realistic supported membranes comprised of microdomains with interstitial liquid-disordered (non-raft) regions.

Can molecular-machine mimics of neural membrane microdomains contribute to more realistic, computationally subtle models in neural networks and artificial intelligence? The answer is guardedly affirmative because, although much has been accomplished, significant engineering obstacles remain [76–81]. The most promising architectures for investigating signal-protein interactions and ion-channel functioning in microdomains are mobile planar lipid bilayers on solid supports such as gold or silicon (Figure 3). Ion-channel insertion, measurable ion flow, and membrane lateral mobility are best achieved by decoupling the membrane from the support by means of a water layer or hydrated polymer. The latter, a biomimic of the cytoskeleton, can stabilize the system for analysis (while sacrificing some lateral mobility) if the polymer layer is covalently tethered to the substrate and attached to the membrane by anchor lipids. Channel-forming proteins have been inserted into engineered membranes with varying levels of biological realism. Traditionally, gramicidin A—a dimer synthesized by binding immobile and mobile half-channels to create a conducting channel—has been widely used as an ion-channel model system. Although this strategy continues, interest in alternative models has been stimulated by the recent successful insertion of functioning glutamate-receptor ion channels (mostly NMDA type) into a mixed hybrid bilayer membrane [82]. Gabriele Favero’s team, who carried out the study, anticipates similar analyses of AMPA and other receptors.

Under more biologically realistic—albeit less stabilized—conditions in which the supported membrane is comprised of a mixture of tethered microdomains and interstitial liquid-disordered regions, it may be possible to investigate the putative NMDA-induced recruitment of AMPA into microdomains (as proposed in Hou’s study), as well as the interactions of other scaffolded signal proteins. Another potentially valuable study would examine microdomain effects on the voltage-gated A-current potassium channel (K_A_). Members of this microdomain-localized channel vary in their kinetics and voltage dependence, but share a critical property: they are transiently activated by depolarization following a period of hyperpolarization [83]. This feature has the important consequence of increasing local hyperpolarization, frequently resulting in AP propagation failure. If K_A_ kinetics in an artificial system can be directionally modulated by the microdomain environment (e.g., via lipid-protein electrostatics as outlined briefly above), this finding would be consistent with a model of neuron modules regulating AP propagation. Studies along these lines could potentially motivate the development of novel architectures (perhaps departing significantly from the traditional McCulloch-Pitts neuron) in neural networks and artificial intelligence.

## 6. Conclusion

Frequently misunderstood by early cell biologists due to flawed instrumentation and inaccurate paradigms, the cell membrane is now widely recognized as an important regulatory structure. The microdomain (or raft) in particular is increasingly viewed as a molecular scaffold which co-localizes signal proteins for orchestrating extra- and intracellular molecular processes. In the special case of the neuron, microdomains appear to modulate AP propagation, synaptic transmission, and a wide variety of cytosolic cascades. Growing evidence therefore supports a revised view of the neuron as an array of computational modules analogous to a chip or integrated circuit. The new viewpoint, if supported by subsequent investigations, may have significant implications for novel artificial-intelligence and neural network designs.
